# *ROS1* copy number alterations are frequent in non-small cell lung cancer

**DOI:** 10.18632/oncotarget.6921

**Published:** 2016-01-15

**Authors:** Sergi Clavé, Javier Gimeno, Ana M. Muñoz-Mármol, Joana Vidal, Noemí Reguart, Enric Carcereny, Lara Pijuan, Sílvia Menéndez, Álvaro Taus, José Luís Mate, Sergio Serrano, Joan Albanell, Blanca Espinet, Edurne Arriola, Marta Salido

**Affiliations:** ^1^ Laboratori de Citogenètica Molecular, Servei de Patologia, Hospital del Mar, Barcelona, Spain; ^2^ Programa de Recerca en Càncer, IMIM (Institut Hospital del Mar de Investigacions Mèdiques), Barcelona, Spain; ^3^ Servei de Patologia, Hospital del Mar, Barcelona, Spain; ^4^ Servei de Anatomia Patològica, Hospital Universitari Germans Trias i Pujol, Badalona, Spain; ^5^ Servei de Oncologia Mèdica, Hospital del Mar, Barcelona, Spain; ^6^ Servei de Oncologia Mèdica, ICMHO, Hospital Clinic Barcelona, Barcelona, Spain; ^7^ Departament de Oncologia Mèdica, Institut Català de Oncologia (ICO), Hospital Universitari Germans Trias i Pujol, Badalona, Spain; ^8^ Cancer Sciences Unit, University of Southampton. Southampton, United Kingdom

**Keywords:** ROS1, FISH, copy number alterations, heterogeneity, IHC

## Abstract

**Objectives:**

We aimed to determine the prevalence and partners of *ROS1* rearrangements, to explore the correlation between FISH and IHC assays, and to investigate clinical implications of *ROS1* copy number alterations (CNAs).

**Methods:**

A total of 314 NSCLC patients were screened using *ROS1* FISH break-apart probes. Of these, 47 surgical tumors were included in TMAs to analyze *ROS1* heterogeneity assessed either by FISH and IHC, and chromosome 6 aneusomy. To characterize *ROS1* partners, probes for *CD74*, *EZR*, *SLC34A2* and *SDC3* genes were developed. *ROS1* positive FISH cases were screened also by IHC.

**Results:**

Five patients were *ROS1* positive (1.8%). We identified two known fusion partners in three patients: *CD74* and *SLC34A2*. Four out of five *ROS1* rearranged patients were female, never smokers and with adenocarcinoma histology. Rearranged cases were also positive by IHC as well. According to *ROS1* CNAs, we found a prevalence of 37.8% gains/amplifications and 25.1% deletions.

**Conclusions:**

This study point out the high prevalence of *ROS1* CNAs in a large series of NSCLC. *ROS1* gains, amplifications and deletions, most of them due to chromosome 6 polysomy or monosomy, were heterogeneous within a tumor and had no impact on overall survival.

## INTRODUCTION

Over the last decade there have been important advances in understanding the biology of non-small cell lung cancer (NSCLC), identifying oncogene-driven subtypes of tumors such as epidermal growth factor receptor (*EGFR*) mutations or anaplastic lymphoma kinase (*ALK*) and ROS proto-oncogene 1 (*ROS1*) rearrangements [1-3]. Targeted therapies based on molecular diagnostics have opened a new era of personalized medicine in NSCLC treatment, and during the last years new predictive biomarkers have emerged [4, 5].

*ROS1* gene (located at chromosome 6q22) encodes for a receptor tyrosine kinase which belongs to the insulin receptor family [6]. In a similar way to *ALK* aberrant kinase activity, rearrangements involving the *ROS1* gene lead to a constitutively activated downstream signaling of several oncogenic pathways [7, 8]. *ROS1* rearrangements are rare events accounting for up to 0.6-1.8% of patients with NSCLC [9, 10]. Until now, several fusion partners (n=12) have been described, being *CD74*, *EZR*, *SLC34A2* and *SDC3* the most prevalent [11, 12]. *ROS1* rearrangements are more commonly found in never or mild smokers patients with adenocarcinoma (ADC) histology and in triple negative (*EGFR*/*KRAS*/*ALK*) population, similar to patients with *ALK*-rearranged NSCLC [13, 14].

Crizotinib (Xalkori®, Pfizer), approved by the US Food and Drug Administration (FDA) in treating *ALK* positive NSCLC, binds also with high affinity to *ROS1-*rearrangedreceptors [15]. In addition to previous case reports [2, 13], a recent study described marked antitumor responses to Crizotinib in patients harboring *ROS1*-rearranged NSCLC [11].

Adequate molecular-based selection is essential for NSCLC patients to achieve optimal results from targeted therapies. Fluorescence in-situ hybridization (FISH) with break-apart probes remains the gold standard method for detecting both *ALK* and *ROS1* rearrangements in lung clinical trials [11, 16]. However, other methods such as immunohistochemistry (IHC) using anti-ROS1 antibodies could provide a widely available alternative screening method. Despite previous manuscripts described a reasonable correlation between FISH and IHC, more information about IHC sensitivity and specificity is needed [17, 18].

In this study, we analyzed a Spanish cohort of NSCLC to determine the prevalence and specific features of *ROS1* rearranged patients and to explore the correlation between FISH and IHC assays. We also investigated *ROS1* copy number alterations (CNAs) to clarify the correlation between *ROS1* gene alteration and clinicopathological parameters.

## RESULTS

### Clinicopathological characteristics

The median age was 64 years, 69% were males and 71% were ever smokers with a median cumulative index of 52 packs per year (Table 1). Fifty-two per cent of the study population presented stage IV disease and 83% were ADC. The predominant histological patterns in ADC were acinar and solid (some of them mucinous type), whereas lepidic and micropapillary patterns were less common. Other histological subtypes were: squamous cell carcinoma (SCC), 3%; large cell carcinoma (LCC), 2%; and non-small cell lung carcinoma not otherwise specified (NSCLC NOS), 12% of patients. Patients with SCC were added in the analysis due to their clinical characteristics (non-smoking patients). *KRAS* and *EGFR* mutations were found in 17% and 6% of the samples respectively, whereas 1% (4 cases) presented *ALK* rearrangements.

**Table 1:** Clinicopathological features of the 314 *ROS1*-screened NSCLC patients

Feature	Global population	*ROS1* positive (*n* = 5)	*p*-value
	*n* (%)		
Median Age: y (range) (*n* = 307)	64 (25-91)	59 (42-74)	0.503
Sex (*n* = 309)			
Male	212 (69)	1 (20)	0.039
Female	97 (31)	4 (80)	
Smoking status (*n* = 287)			
Non smoker	82 (29)	4 (80)	0.030
Ever smoker	205 (71)	1 (20)	
Stage (*n* = 284)			
I	63 (22)	0	0.646
II	23 (8)	0	
III	51 (18)	1 (20)	
IV	147 (52)	4 (80)	
Histology (*n* = 309)			
ADC^1^	256 (83)	4 (80)	0.827
SCC^2^	11 (3)	0	
LCC^3^	6 (2)	0	
NSCLC NOS^4^	36 (12)	1 (20)	
*KRAS* (*n* = 210)			
Wild-type	174 (83)	5 (100)	1.000
Mutated	36 (17)	0	
*EGFR* (*n* = 276)			
Wild-type	260 (94)	5 (100)	1.000
Mutated	16 (6)	0	
*ALK* (*n* = 311)			
Non-rearranged	309 (99)	5 (100)	1.000
Rearranged	4 (1)	0	

1 Adenocarcinoma

2 Squamous cell carcinoma

3 Large cell carcinoma

4 Non-small cell lung carcinoma not otherwise specified

### *ROS1* rearrangements

**Figure 1:**
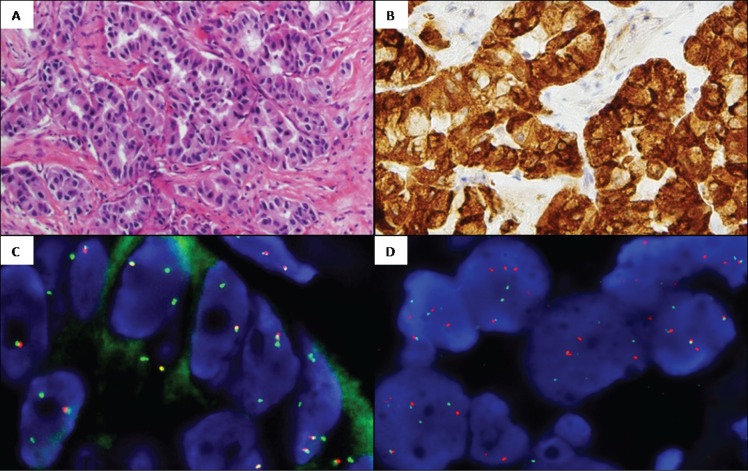
Representative images from case 2 *ROS1*-rearranged: **A.** Hematoxylin and eosin, 20x. Adenocarcinoma showing a predominantly acinar pattern. **B.** ROS1 IHC, 20x. Cytoplasmatic +3/+4 staining pattern. **C.** *ROS1* FISH, 100x. Break-apart probe showing an atypical rearranged pattern with *5′ROS1* deletions (1F2G). **D.** CD74 FISH, 100x. Break-apart probe showing a typical rearranged pattern with fused and split signals (2F2O2G).

*ROS1* FISH was evaluable in 283 cases (90.1%). The basket cases were due to insufficient tumor material or FISH assay failure. *ROS1* rearrangements were found in five cases (1.8%); three of them showed a typical rearranged pattern with deletion of the fused allele (1O1G) and the other two cases had an atypical rearranged pattern with *5′ROS1* deletion (1F1G) and with gains (1F2G) (Figure 1). Of note, one of the typical rearranged cases was heterogeneous showing a negative area with *ROS1* deletion. The mean percentage of positive cells in all cases was 70%. Regarding to *ROS1* partners, they were identified in three cases: two harbored a t(5;6)(q32;q22)with *CD74*-*ROS1* fusion, one of them showing isolated 5′*CD74* signals; and the third presented a t(4;6)(q15.2;q22)with *SLC34A2*-*ROS1* rearrangement.

Four of these five tumors presented ADC histology, the other one classified as NSCLC NOS. Two of the confirmed ADC had a predominantly solid pattern, whereas the two remaining cases had a predominantly papillary pattern. Regarding to ROS1 IHC pattern of staining, three out of the five cases presented a cytoplasmic predominant pattern whereas only one case presented a membranous predominant pattern (with mild +1 cytoplasmic associated ROS1 staining). Material was not available from the last case to perform ROS1 IHC assay. Characteristics of *ROS1* positive cases are presented in Table 2.

**Table 2:** Histopathological features of *ROS1*-rearranged specimens

Case	FISH pattern^1^	Fusion partner	H-Score	Staining pattern	Sample	Histology, predominant
*ROS1* 1	1O1G	*SLC34A2*	400	Cytoplasmic	Visceral pleura	NSCLC NOS^2^, N/A^3^
*ROS1* 2	1F2G	*CD74*	300-400	Cytoplasmic	Lung	ADC^4^, acinar
*ROS1* 3	1O1G	N/A	400	Membranous	Lung	ADC, solid
*ROS1* 4	1O1G	*CD74*	300-400	Cytoplasmic	Lung	ADC, papillary
*ROS1* 5^5^	1F1G	N/A	N/A	N/A	Lung	ADC, papillary

1 FISH pattern indicates the result of the *ROS1* break-apart probe used, labeled 5′ROS1 Spectrum orange (O) and 3′*ROS1*Spectrum green (G).

2 Non-small cell lung carcinoma not otherwise specified

3 Not available

4 Adenocarcinoma

5 Material was not available to perform FISH fusion partner studies and ROS1 IHC assay.

We observed a significant association between *ROS1* rearrangement and female gender (*p*=0.039) as well as with non-smoking history (*p*=0.030).

### *ROS1* copy number alterations

**Figure 2:**
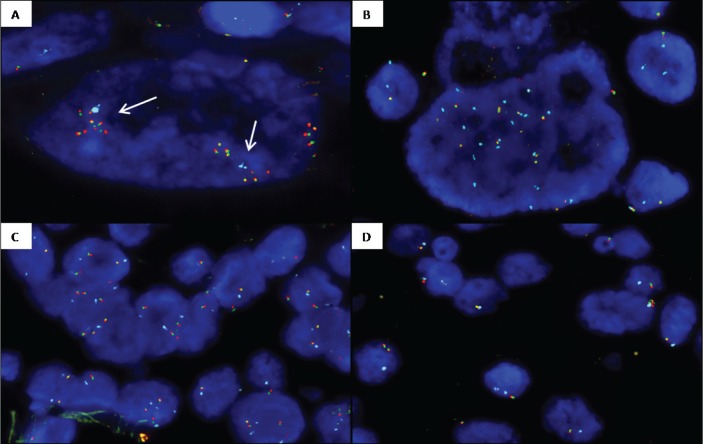
Representative images from *ROS1* copy number alterations detected by FISH (*ROS1* break-apart probe (spec.orange/green fusion signals) and CEP6 probe (spec.aqua signals) **A.** *ROS1* cluster amplification (arrows) in a nuclei with two copies of CEP6. **B.** *ROS1* amplification with chromosome 6 high polysomy (15 *ROS1* and 15 CEP6 copies). **C.** Polysomic nuclei with 3-4 copies of chromosome 6 and 3-4 copies of *ROS1*. **D.** Case showing heterogeneity of *ROS1* deletion: nuclei with *ROS1* monosomy (1 *ROS1* 1 CEP6) and nuclei with *ROS1* deletion and chromosome 6 disomy (1 *ROS1*/2 CEP6).

In *ROS1* non-rearranged cases, gains were the most prevalent CNAs observed in 96 cases (33.9%) and in a high proportion of cells (mean percentage of 48%). Using CEP6 control probe, we confirm that gain of *ROS1* was due to chromosome 6 polysomy. *ROS1* amplifications were identified in eleven cases (3.9%), all of them with ADC histology. Amplified cases were classified in two groups: patients that had *ROS1* signal clusters (n=3) and patients with a chromosome 6 high polysomy (n=8) (Figure 2). In addition, 71 cases (25.1%) had *ROS1* deletions with a mean percentage of *ROS1* deleted cells of 42%. After CEP6 hybridization, heterogeneity in the enumeration of chromosome 6 was observed. Both monosomy and polysomy of CEP6 were observed in the same tumor sample.

All the specimens that were negative for the *ROS1* rearrangements by FISH presented negative IHC ROS1 staining. In some of them, mild (+1/+2) and diffuse cytoplasmic staining was detected (presenting H-score: <200). If only 3+ (diffusely) expressing tumors were considered positive, ROS1 IHC is 100% sensitive and 87.5% specific for the presence of *ROS1* rearrangements by FISH. Of note, in some cases mild (+1) ROS1 cytoplasmic staining was detected in benign type II pneumocytes.

### Heterogeneity assessment

**Figure 3:**
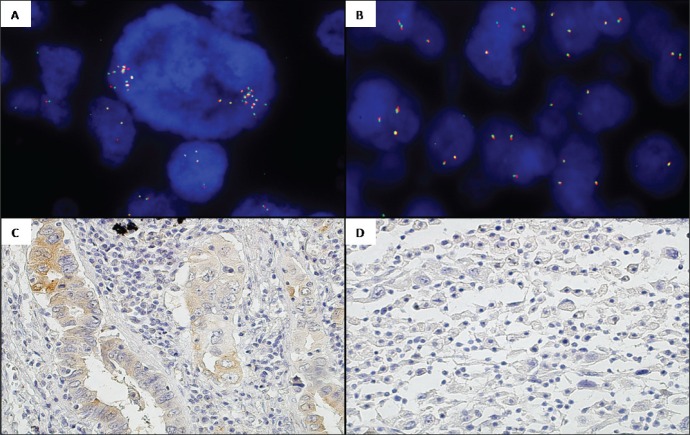
Intra-tumor heterogeneity regarding *ROS1* status by FISH and IHC in one TMAs case **A.** *ROS1* FISH, 100x. Core 1: Negative *ROS1* presenting cells with focal amplifications. **B.** *ROS1* FISH, 100x. Core 2: Disomic *ROS1* cells and nuclei with *ROS1* gene gains without amplification. **C.** ROS1 IHC, 20x. Core 1: ROS1 +1/+2 predominantly acinar. **D.** ROS1 IHC, 20x. Core 2: ROS1 0 discohesive (sarcomatoid).

Focusing on heterogeneity in TMAs, 161 out of 192 cores were assessable for *ROS1* FISH analysis and 174 for ROS1 IHC (Figure 3). As no positive *ROS1* cases were found in the TMAs, the heterogeneity assessment was performed considering only *ROS1* CNAs. When evaluating CNAs as a categorical variable (gain, amplification, deletion and disomic), Kappa agreement index for *ROS1* FISH status between cores A and B was 0.24. When ROS1 IHC status was analyzed considering 0, +1, +2 and +3 score criteria, no differences in classification between cores A and the remaining three cores (B to D) were observed. The result changed from negative to borderline +2 only in four cases.

### Survival analysis

**Figure 4:**
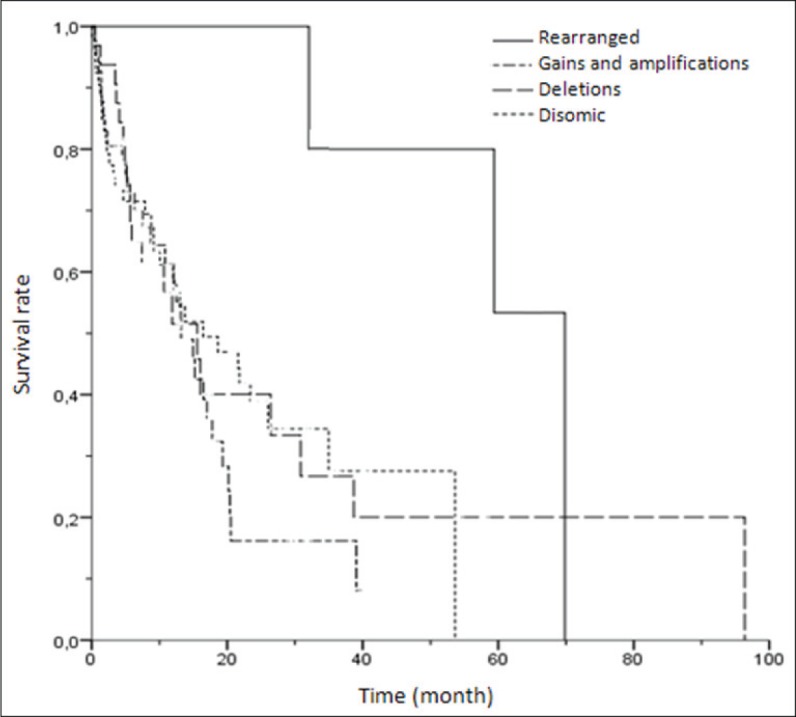
Kaplan-Meier univariate analysis of survival rates among patients with different *ROS1* FISH results (rearranged, gains/amplifications, deletions and disomic cases) (*p* = 0.087)

We sought to explore the potential impact on survival of *ROS1* alterations, analyzed only in patients with advanced disease (stages III-IV). Out of 144 cases with available data, 94 death events occurred during the follow-up period. The median follow-up time for the whole series was 15.2 months (95% CI 12-18.3) and median survival time was 26.2 month. One, two and three-year survival rates were 59.1%, 33% and 21.6%, respectively. The median survival time in *ROS1*-positive patients was significantly higher: 69.8 month vs. 13.7 months (95% CI 9.9-17.5) in the *ROS1*-negative group (*p*=0.028) (Figure 4). All positive patients received Crizotinib treatment. Setting survival analysis according to CNAs, neither copy number gains nor deletions of *ROS1* gene had impact on survival.

## DISCUSSION

We examined *ROS1* status in a cohort of patients with NSCLC. The main findings of our study are the following: (1) the prevalence of *ROS1* gene rearrangement in a Spanish cohort is 1.8%; (2) *ROS1*-rearranged cases are significantly associated with female gender and never smoker patients; (3) IHC correlates with FISH in *ROS1*-rearranged cases; (4) *ROS1* CNAs are frequent, with a remarkably 33.9% of gains, 3.9% of amplifications, and 25.1% of deletions; (5) *ROS1* CNAs had no impact on overall survival.

In previous studies, *ROS1* rearrangements were detected in 0.8 to 2.5% of unselected NSCLC patients [12, 19]. Here we present a cohort of cases evaluated for *ROS1* status using FISH that confirms the low frequency of *ROS1* rearrangements in a Caucasian population. Similar to prior reports, we found an association of *ROS1* rearrangement with non-smoking history [19, 20]. Moreover our study reports an association with female patients. The clinical profile of these patients is remarkably similar to that of *ALK*-rearranged, including features like gender and non-smoking history [13] and also advanced stage [21]. As recent publications demonstrate, the frequency of *ROS1* rearrangements in clinically selected patients is higher, reaching values of 7.4% [18]. Giving that fact, a properly clinical selection can significantly enrich for the incidence of *ROS1* rearrangements in the tested population.

*ROS1* rearranged cases were detected also with ROS1 D4D6 IHC. In previous studies, FISH and IHC had a good correlation although optimal IHC criteria need to be established [9, 17]. As other authors mentioned, *ROS1* is expressed in NSCLC without concomitant translocation [22] and also some cases with *ROS1* rearrangements detected by FISH have low protein expression suggesting a low sensitivity of IHC [9, 23]. In our hands, IHC analysis optimally detects *ROS1* positive cases although additional studies are needed to consider this method as a screening routine test.

Regarding FISH characterization of positive cases, we identify two known fusion partners: *CD74* and *SLC34A2*. Clinically, the small numbers of cases preclude any correlation between the specific fusion partner and outcome [12, 24] as well as to date, no correlation has been observed between the type of *ROS1* rearrangement and clinical response to Crizotinib [11]. Next generation sequencing (NGS) techniques could be a better approach to identify all *ROS1* fusion genes, due to the high number of fusion partners described. Still, the main limitation of these techniques is to have sufficient material to be studied [25].

Regarding *ROS1* CNAs, we found a high prevalence of *ROS1* gene copy number gains without impact in overall survival. Jin et al. (2015) recently reported that *ROS1* gains were significantly associated with both shorter disease-free survival and shorter overall survival [19]. It is remarkable that they only found 4.8% of cases with *ROS1* gains whereas in our cohort the percentage of samples with gains/amplifications was significantly higher (37.8%). They studied unselected cohort of surgically resected NSCLC patients, and found that copy number gains are more common in male patients with squamous histology. Most of the patients included in our study had a stage III-IV (70%) with a low representation of squamous histology (3%). Differences in patient selection could explain discrepancies. In our series, amplifications were identified in only eleven specimens, all of them presenting signal clusters or chromosome 6 high polysomy in large cells. Remarkably, ROS1 protein expression was not detectable in any of these cases suggesting that amplification might not be a biologically relevant event or predict response to *ROS1* inhibitors [26].

Additionally, the current study demonstrates that *ROS1* gene copy number is heterogeneous within surgical NSCLC specimens. This finding has potential diagnostic implications considering that in our routine clinical practice endoscopic, core-needle biopsies or cytology samples containing only a small part of tumor were used to make treatment decisions.

Interestingly, our study is the first to report a high prevalence of *ROS1* deletions. Heterogeneity in the enumeration of chromosome 6 was observed, being 6q deletion or monosomy or both, the cause of *ROS1* loss. Previous cytogenetic studies demonstrated that loss of 6q, where *ROS1* gene is located, is one of the commonest chromosomal aberrations in lung ADC [27, 28]. So, as shown in our cohort, it is expected to observe *ROS1*-positive samples with concomitant deletion of *ROS1* non-rearranged allele. Particularly, one of the *ROS1* positive tumors exhibits two distinct areas, one with *ROS1* gene deletion without rearrangement and the other with positive *ROS1* 1O1G pattern suggesting that even though *ROS1* rearrangements are driver mutations, 6q deletion/monosomy could occur as a primary event in NSCLC. Opposed to the other *ROS1* positive patients that elicited a partial response to Crizotinib, this patient did not respond, suggesting that heterogeneity of genetic alterations could explain differences in response to treatments.

Our study demonstrates a significant increased median overall survival time for *ROS1*-positive compared to *ROS1*-negative NSCLC patients (69.8 vs. 13.7 month) (*p*=0.028). This result is consistent with recently data reported by Scheffler et al. (2015): *ROS1* seems to be one of the best prognostic molecular markers in NSCLC and stays in line of recent reports pointing the high efficacy of Crizotinib treatment for these patients [29, 30].

In conclusion, our study confirms the low prevalence of *ROS1* rearrangements in Spanish NSCLC patients and shows a high prevalence of *ROS1* CNAs that should be taken into account when assessed by FISH although do not present associated clinical implications. *ROS1* break-apart FISH is herein validated as a reliable method to diagnose *ROS1* rearrangements and also as a detailed method that helps us to understand the biology of NSCLC tumors more deeply.

## MATERIAL AND METHODS

### Study population

A total of 314 formalin-fixed and paraffin-embedded (FFPE) samples referred between 2012 and 2014 were available for this study. Material confirmed for NSCLC (surgical specimens, core-needle biopsies or cytological cell-blocks) was screened for *ROS1* rearrangements using FISH in two Spanish centers: Hospital del Mar, Barcelona (n=247), and Hospital Germans Trias i Pujol, Badalona (n=67). *ROS1* FISH was performed in sequence to *EGFR*, *KRAS* and *ALK* testing. This project was approved by the local ethics committee (CEIC-IMAS) and all patients provided written informed consent. Clinical data were extracted from medical records and included age, sex, smoking history, tumor disease stage and clinical follow-up information.

### Tissue microarray construction

Based on tissue availability, 47 surgically resected tumors from our Institution were selected to construct three tissue microarrays (TMAs) [31]. Briefly, original hematoxylin and eosin-stained sections were reviewed from each patient to determine different malignant areas and to identify benign lung tissue. Three tissue cores were obtained from each patient, two of them containing different histological areas of the carcinoma (named A and B) and the third containing benign lung parenchyma. The cores then were brought into a new recipient and a paraffin block was constructed using a tissue micro arrayer (Beecher Instruments, Sun Prairie WI). To assess heterogeneity three other TMAs were constructed with different replicas of each tumoral area (named C and D).

### ROS1 fluorescence *in situ* hybridization (FISH)

FISH was conducted on FFPE tissues from the whole tumor samples and the TMAs sections as previously described [32], using commercially break-apart *ROS1* locus probes (Abbott Molecular, Des Plaines, IL and/or ZytoVision, Bremerhaven, Germany). A minimum of fifty non-overlapping cells with hybridization signals were examined for each case with a BX51 fluorescence microscope (Olympus, Tokyo, Japan). Samples were considered positive if more than 15% of cells showed split 5′*ROS1* (Spec. Orange) and 3′*ROS1* (Spec. Green) signals (typical rearranged pattern) or isolated *3′ROS1* signals (atypical rearranged pattern). The FISH rearrangement patterns are described in the text as the number of fusion signals (F) followed by the number of 5′*ROS1* signals (O) and 3′*ROS1* signals (G). *ROS1* CNAs such as gains/amplifications and deletions were also recorded. Gain was defined as a mean copy number greater than 3 fused signals in >40% of nuclei, and amplification as the presence of >15 copies of *ROS1* per cell (high polysomy) or clusters, in a minimum of 15% of analyzed cells. Cut-off for *ROS1* deletion was assessed in benign lung parenchyma from TMAs cores and established at 1 copy in >30% of nuclei. Moreover, in order to determine the cause of *ROS1* CNAs, TMAs and *ROS1* amplified cases were analyzed with CEP6 control probe (Spec. Aqua; Abbott Molecular, Des Plaines, IL).

Positive cases were screened for the detection of fusion partners using break-apart non-commercial probes selected from the Human 32K BAC Re-Array Library (BACPAC Resource Center, Oakland, CA). The following clones were selected: *CD74* (5q32): RP11-725L10 and RP11-690J03; *EZR* (6q25.3): RP11-654E18 and RP11-268N15; *SLC34A2* (4p15.2): RP11-659J14 and RP11-790A19; and *SDC4* (20q12): RP11-69D17 and RP11-220B01.

### ROS1 immunohistochemistry (IHC)

ROS1 IHC was performed using the commercially available clone D4D6 (Cell Signalling Technology, Danvers, MA) on unstained FFPE tissue sections from the whole tumor of *ROS1* positive and the TMAs samples. The primary antibody was manually applied at 1:50 dilution and incubated at 37°C for 1 hour. Samples were then revealed using the FlexPlus DAB Detection Kit in a Dako Autostainer Plus (Dako, Carpinteria, CA). ROS1 IHC staining was evaluated by two independent pathologists. Staining was graded semiquantitatively as follows: 0 for absent expression or nuclear expression only, 1+ for cytoplasmic faint, barely perceptible staining not exceeding background in any percentage of cells, 2+ for cytoplasmic staining exceeding background in 0 to 50% of tumor cells, and 3+ for cytoplasmic staining exceeding background in >50% of tumor cells [33]. Moreover, H-score was recorded as initially described to evaluate EGFR expression [34]. Briefly, this score ranges from 0 to 400 that result from the combination of the staining intensity (0-4) and the percentage of positive tumor cells (0-100%) in each sample.

### Statistical analysis

The whole series of cases were studied in order to characterize associations between clinicopathological variables and FISH data (*ROS1* rearrangement and *ROS1* CNAs). From TMAs cases, the core with the most prevalent CNA was selected. Statistical associations were assessed using Pearson's χ2 test or Fisher's exact test, depending on the sample size. All statistical tests were conducted at the two-sided 0.05 alpha level of significance. Survival curves were obtained with the Kaplan-Meier method, and log-rank test was used for comparison of survival curves between different groups of patients. For each patient, cumulative survival was calculated from the date of diagnosis to death (from any cause) or last follow-up. Heterogeneity of FISH and IHC data of TMAs was assessed using Kappa agreement index for categorical variables. All statistical analyses were carried out with SPSS version 15 (SPSS Inc., Chicago, IL).
